# The Association of Peroxisome Proliferator-Activated Receptor *δ* and Additional Gene-Gene Interaction with C-Reactive Protein in Chinese Population

**DOI:** 10.1155/2016/8597085

**Published:** 2016-01-13

**Authors:** Xiao-Ying Ding, Hao-Zheng Yuan, Ru Gu, Yan-Feng Gao, Xiao-Gang Liu, Ya Gao

**Affiliations:** ^1^Department of Anesthesia, The Second Affiliated Hospital, School of Medicine, Xi'an Jiaotong University, Xi'an, Shaanxi 710004, China; ^2^Department of Anesthesia, The First Affiliated Hospital, School of Medicine, Xi'an Jiaotong University, Xi'an, Shaanxi 710061, China; ^3^The Key Laboratory of Biomedical Information Engineering of Ministry of Education, School of Life Science and Technology, Xi'an Jiaotong University, Xi'an, Shaanxi 710049, China; ^4^Department of Pediatric Surgery, The Second Affiliated Hospital, School of Medicine, Xi'an Jiaotong University, Xi'an, Shaanxi 710004, China

## Abstract

*Aims*. To examine the association between 4 single nucleotide polymorphisms (SNPs) of peroxisome proliferator-activated receptors *δ* (PPAR*δ*) polymorphisms and C-reactive protein (CRP) level and additional gene-gene interaction.* Methods*. Line regression analysis was performed to verify polymorphism association between SNP and CRP levels. Generalized multifactor dimensionality reduction (GMDR) was employed to analyze the interaction.* Results*. A total of 1028 subjects (538 men, 490 women) were selected. The carriers of the C allele (TC or CC) of rs2016520 were associated with a significant decreased level of CRP, regression coefficients was −0.338, and standard error was 0.104 (*p* = 0.001). The carriers of the G allele (CG or GG) of rs9794 were also significantly associated with decreased level of CRP, regression coefficients was −0.219, and standard error was 0.114 (*p* = 0.012). We also found a potential gene-gene interaction between rs2016520 and rs9794. Subjects with rs2016520-TC or CC, rs9794-CG or GG genotypes have lowest CRP level, difference (95% CI) = −0.50 (−0.69 to −0.21) (*p* < 0.001), compared to subjects with rs2016520-TT and rs9794-CC genotypes.* Conclusions*. rs2016520 and rs9794 minor allele of PPAR*δ* and combined effect between the two SNP were associated with decreased CRP level.

## 1. Introduction

Inflammation is receiving an increasing amount of attention for its potential role in the pathogenesis of a variety of disorders, from insulin resistance and type 2 diabetes mellitus (T2DM) to fatty liver and cardiovascular disease (CVD) [1]. Current evidence shows that C-reactive protein (CRP) level is a plasma protein and a sensitive and dynamic systemic marker of inflammation [2] could be influenced by both clinical and genetic factors [3].

PPARs are orphan nuclear receptors belonging to the steroid, retinoid, and thyroid hormone receptor superfamily of ligand-activated transcription factors [4, 5]. Three distinct receptor types have been cloned and characterized: PPAR*α*, PPAR*β*/*δ*, and PPAR*γ* [6]. PPAR*δ* was less studied in previous study, compared with PPAR*α* and PPAR*γ*. Several evidences suggested biological roles for PPAR*δ* in many genotypes [7]. Deletion of PPAR*δ* from foam cells increased the availability of inflammatory suppressors, which in turn reduced atherosclerotic lesions [8]. On the other hand, activation of PPAR*δ* stimulates *β*-oxidation and triglyceride utilization in adipocytes and myocytes. PPAR*δ* agonists could promote cholesterol accumulation in human macrophages and increase serum high density lipoprotein (HDL) while lowering triglyceride levels in obese animal models [9, 10]. These data collectively implicate PPAR*δ* as being involved in lipid homeostasis and atherogenesis. However, the associations between variants of the PPAR*δ*, corresponding gene-gene interactions with CRP level, were rarely studied. So in this study, we sought to examine the association between 4 PPAR*δ* polymorphisms with CRP level and the additional interaction among the 4 single nucleotide polymorphisms (SNPs).

## 2. Materials and Methods

### 2.1. Subjects

This was a cross-sectional study. Chinese participants were consecutively recruited between January 2011 and September 2013. A total of 1096 subjects were included in investigated population, excluded subjects with abnormal increasing of CRP level (≥10 mg/L) because of any reasons, such as infection (*n* = 35), trauma (*n* = 15), and any others factor (*n* = 8), and CRP were missing (*n* = 10), a total of 1028 subjects (538 men, 490 women), with a mean age of 45.3 ± 13.8 years, were included in the study, including the genotyping of polymorphisms. The selected subjects were similar to those who were not selected in terms of age, sex, smoking status, and alcohol consumption. Informed consent was obtained from all participants.

### 2.2. Body Measurements

Data on demographic information and lifestyle risk factors for all participants were obtained using a standard questionnaire administered by trained staffs. Body weight and height were measured, and body mass index (BMI) was calculated as weight in kilograms divided by the square of the height in meters. Cigarette smokers were those who self-reported smoking cigarettes at least once a day for 1 year or more [11]. Alcohol consumption was expressed as the sum of milliliters of alcohol per week from wine, beer, and spirits [12]. Physical activity was divided into three levels, including light, moderate, and heavy [13]. Blood samples were collected in the morning after at least 8 hours of fasting. All plasma and serum samples were frozen at –80°C until laboratory testing. Plasma glucose was measured using an oxidase enzymatic method. Particle enhanced immune latex agglutination high sensitive assay was used to detect CRP level. All analysis was performed by the same lab.

### 2.3. Genomic DNA Extraction and Genotyping

We selected SNPs within the PPAR*δ* gene using the following methods: (1) previously reported associations with metabolic abnormalities, (2) known heterozygosity and a minor allele frequency (MAF) greater than 1%. 4 SNPs of PPAR*δ* were selected for genotyping in the study: rs2016520, rs9794, rs1053046, and rs1053049. Genomic DNA from participants was extracted from EDTA-treated whole blood, using the DNA Blood Mini Kit (Qiagen, Hilden, Germany) according to the manufacturer's instructions. All SNPs were detected by Taqman fluorescence probe. Probe sequences of all SNPs were shown in Table 1. ABI Prism7000 software and allelic discrimination procedure were used for genotyping of aforementioned 4 SNPs. A 25 *μ*L reaction mixture including 1.25 *μ*L SNP Genotyping Assays (20x), 12.5 *μ*L Genotyping Master Mix (2x), and 20 ng DNA and the conditions were as follows: initial denaturation for 10 min and 95°C, denaturation for 15 s and 92°C, annealing and extension for 90 s and 60°C, and 50 cycles.

### 2.4. Statistical Analysis

The mean and standard deviation (SD) for normally distributed continuous variables and percentages for categorical variable were calculated and compared. The genotype and allele frequencies were obtained by direct count. The categorical data were analyzed using *χ* ^2^ test or the Fisher exact test if necessary. Further, continuous variables were analyzed using Student's *t*-test or one-way analysis of variance, followed by the least significant difference multiple-range tests for comparison between groups. Hardy-Weinberg equilibrium (HWE) was performed by using SNPStats (available online at http://bioinfo.iconcologia.net/SNPstats). Line regression analysis was performed to verify polymorphism association between SNP with CRP levels using gender, age, smoking and alcohol status, and physical activity as covariates in the model.

Generalized multifactor dimensionality reduction (GMDR) [14] was employed to analyze the interaction among 4 SNPs; some parameters was calculated, including cross-validation consistency, the testing balanced accuracy, and the sign test, to assess each selected interaction. The cross-validation consistency score is a measure of the degree of consistency with which the selected interaction is identified as the best model among all possibilities considered. The testing balanced accuracy is a measure of the degree to which the interaction accurately predicts case-control status with scores between 0.50 (indicating that the model predicts no better than chance) and 1.00 (indicating perfect prediction). Finally, a sign test or a permutation test (providing empirical *p* values) for prediction accuracy can be used to measure the significance of an identified model.

## 3. Results

A total of 1028 subjects (538 men, 490 women), with a mean age of 45.3 ± 13.8 years, were selected. Minor allele frequencies of rs2016520, rs9794, rs1053046, and rs1053049 were shown in Table 2. All genotypes were distributed according to Hardy-Weinberg equilibrium (all *p* values more than 0.05).

Line regression analysis on association between 4 SNPs and CRP level was conducted (Table 3). We found that the CRP level was lower in the subjects with minor alleles of rs2016520 and rs9794, compared to subjects with wild genotype. Compared to the carriers of the common genotype (rs2016520-TT), the carriers of the C allele (TC + CC) of rs2016520 were associated with a significant decreased level of CRP, regression coefficients were −0.338, and standard error was 0.104 (*p* = 0.001). Compared to the carriers of the common genotype (rs9794-CC), the carriers of the G allele (CG + GG) of rs9794 were also significantly associated with decreased level of CRP, regression coefficients were −0.219, and standard error was 0.114 (*p* = 0.012). However, the other 2 SNPs in PPAR*δ* were not significantly associated with CRP level after covariate adjustment.

We employed the GMDR analysis to assess the impact of the interaction among 4 SNPs, after adjustment for covariates including gender, age, smoke and alcohol status, and physical activity.  Table 4 summarizes the results obtained from GMDR analysis for two- to four-locus models with covariates adjustment. There was a significant two-locus model (*p* = 0.0107) involving rs2016520 and rs9794, indicating a potential gene-gene interaction between rs2016520 and rs9794. Overall, the two-locus models had a cross-validation consistency of 10 and had the testing accuracy of 62.17%.

To obtain ORS and 95% CIs for the joint effects of candidate SNPs (rs2016520 and rs9794) on CRP, we conducted interaction analysis among SNPs in the 2-locus models. Subjects with rs2016520-TC or CC and rs9794-CG or GG genotypes have lowest CRP level, difference (95% CI) = −0.50 (−0.69 to −0.21) (*p* < 0.001), compared to subjects with rs2016520-TT and rs9794-CC genotypes, after adjustment for gender, age, smoke and alcohol status, and physical activity (Table 5).

## 4. Discussion

The frequency of the C allele was 24.9% in the present population, which is lower than the proportion in the Han population of Dalian reported by Yu et al. [15], similar to that in Korean populations [16], Swedish populations [17], and Scotland populations [18]. C-reactive protein (CRP) is a well-established biochemical marker of inflammation and has been used to predict future cardiovascular disease, metabolic syndrome, and future development of type 2 diabetes mellitus [3, 19–21]. Current evidence shows that CRP level is a complex trait, influenced by both clinical and genetic factors [3]. Family and twin studies have found that additive genetic factors account for 27–40% of the variance in CRP level [22], suggesting a role for genetic variation in determining serum levels. So it is meaningful to investigate mutation risk factors for level. The results of this study indicated that minor alleles of rs2016520 and rs9794 were associated with lower CRP level, compared to subjects with wild genotype. Compared to the carriers of the common genotype (rs2016520-TT), the carriers of the C allele (TC + CC) of rs2016520 were associated with a significant decreased level of CRP. Compared to the carriers of the common genotype (rs9794-CC), the carriers of the G allele (CG + GG) of rs9794 were also significantly associated with decreased level of CRP. However, the other 2 SNPs in PPAR*δ* were not significantly associated with CRP level after covariate adjustment. In contrast to PPAR*γ* and PPAR*α*, the role of PPAR*δ* in CRP level is less well defined. Gu et al. [23] indicated that rs2016520 in PPAR*δ* was not associated with CRP level both in normal weight and obese subjects in a Chinese population. Liang et al. [24] suggested that PPAR*δ* attenuated CRP-induced proinflammatory effects through CD32 and NF-*κ*B pathway. PPAR*δ* may serve as a more potent therapeutic target than PPAR*γ* in atherosclerosis or inflammatory therapy, and the potency of 1 *μ*M PPAR*δ* is similar to that of 10 *μ*M PPAR*γ* in anti-inflammatory effect.

As we have known that a multiple genotype was influenced by both genetic and environment factors and the genetic factors including many gene mutations, so it is necessary to investigate the synergistic effect of multiple SNP on CRP level. We employed the GMDR analysis to assess the impact of the interaction among the 4 SNPs on CRP level with covariates adjustment. The results indicated a potential gene-gene interaction between rs2016520 and rs9794. Subjects with rs2016520-TC or CC and rs9794-CG or GG genotypes have lowest CRP level, difference (95% CI) = −0.50 (−0.69 to −0.21) (*p* < 0.001), compared to subjects with rs2016520-TT and rs9794-CC genotypes. In addition, rs9794 was not associated with CRP, but it can significantly affect level when accompanied with rs2016520. These findings indicate that a minor gene (even when its main effects are close to nil) can have a strong effect on level, due to the presence of gene-gene interaction.

Several mechanisms of association between activation of PPAR*δ* and decreased CRP level have been suggested. Liang et al. [24] indicated that untreated endothelial cells express a basal level of PPAR*δ* and PPAR*δ* agonist exerts an anti-inflammatory effect in CRP-treated endothelial cells. We also demonstrated that less concentration of PPAR*δ* than PPAR*γ* agonist is needed to attenuate both CRP-induced adhesion molecules and the ability of monocyte to attach to endothelial cells. PPAR*δ* could attenuate CRP-induced NF-*κ*B activation and VCAM-1 and MCP-1 expression in endothelial cells. Moreover, it was found that the decreased adhesion molecule expression would result in significant fewer monocytes attaching on the CRP-treated endothelial cells [25]. Similar to one of the mechanisms in endothelial cells, activation of PPAR*δ* has been shown to promote the binding of inflammatory suppressor BCL-6 to the promoter of group IIA secretory phospholipase A2, an inflammatory marker for atherosclerosis, and therefore inhibit the inflammation induced by IL-1b in VSMCs [25].

Limitations of this study should be considered. Firstly, only 4 SNPs of PPAR*δ* were chosen. The selected SNPs were not sufficient to capture most genetic information of the PPAR*δ*. Further studies should include more SNPs, even the other PPAR isoforms, such as PPAR*α* or PPAR*γ*. Secondly, there was a relatively small sample size in the study, though the number of study participants met the requirement for analysis; future studies should be conducted in different races. Thirdly, the interaction between gene-environment factors on level should be studied in the future papers.

In conclusion, the results of this study indicated that minor alleles of rs2016520 and rs9794 were associated with lower CRP level, compared to subjects with wild genotype, and we also found a potential gene-gene interaction between rs2016520 and rs9794 on decreased CRP level.

## Figures and Tables

**Table 1:** Description and probe sequence for 4 SNPs used for Taqman fluorescence probe analysis.

SNP ID	MAF	Chromosome	Functional consequence	Nucleotide substitution	Probe sequence
rs2016520	0.2290	6:35411001	Intron variant, UTR variant 5 prime	T > C	5′-CGGCCACATGCCGCGTCCCTGCCCC[C/G] ACCCGGGTCTGGTGCTGAGGATACA-3′

rs9794	0.1416	6:35428018	UTR variant 3 prime	C > G	5′-CCTCTGCCCAGGCTGATGGGAACCA[C/T] CCTGTAGAGGTCCATCTGCGTTCAG-3′

rs1053046	0.1681	6:35427801	UTR variant 3 prime	A > G	5′-GTCCTCCCTCCCAAGGAGCCATTCT[A/G] TGTGTGACTCTGGGTGGAAGTGCCC-3′

rs1053049	0.3359	6:35427841	UTR variant 3 prime	T > C	5′-TGGAAGTGCCCAGCCCCTGCCCCTA[C/T] GGGCGCTGCAGCCTCCCTTCCATGC-3′

**Table 2:** The genotype and allelic frequencies distribution in the 1028 subjects.

SNPs	Genotypes and alleles	Frequencies *N* (%)	HWE test
rs2016520	TT	590 (57.4)	0.113
	TC	365 (35.5)	
	CC	73 (7.1)	
	T	1545 (75.1)	
	C	511 (24.9)	

rs9794	CC	596 (58.0)	0.320
	CG	381 (37.1)	
	GG	51 (5.0)	
	C	1573 (76.5)	
	G	483 (23.5)	

rs1053046	AA	598 (58.2)	0.703
	AG	375 (36.5)	
	GG	55 (5.3)	
	A	1571 (76.4)	
	G	485 (23.6)	

rs1053049	TT	602 (58.6)	0.533
	TC	374 (36.4)	
	CC	52 (5.0)	
	T	1578 (76.8)	
	C	478 (23.2)	

**Table 3:** Line regression analysis on association between 4 SNPs and CRP level.

SNP ID	Genotypes	CRP (mg/L)	Regression coefficients	Standard error	*p* values^a^
rs2016520	TT	1.32 ± 1.58	−0.338	0.104	0.001
	TC + CC	0.71 ± 1.62			

rs9794	CC	1.28 ± 1.62	−0.219	0.114	0.012
	CG + GG	0.65 ± 1.70			

rs1053046	AA	1.08 ± 1.59	−0.098	0.146	0.502
	AG + GG	0.94 ± 1.79			

rs1053049	TT	1.06 ± 1.45	−0.146	0.105	0.164
	TC + CC	0.89 ± 1.86			

^a^Adjusted for gender, age, smoke and alcohol status, and physical activity.

**Table 4:** Best gene-gene interaction models, as identified by GMDR.

Locus number	Best combination	Cross-validation consistency	Testing accuracy	*p* values^a^
2	rs2016520 rs9794	10/10	0.6217	0.0107
3	rs2016520 rs9794 rs1053049	9/10	0.5577	0.1719
4	rs2016520 rs9794 rs1053046 s1053049	9/10	0.5590	0.0547

^a^Adjusted for gender, age, smoke and alcohol status, and physical activity.

**Table 5:** Interaction analysis for two-locus models.

rs9794	rs2016520	Difference (95% CI)^a^	*p* values
CC	TT	0.00	—
CG or GG	TT	0.29 (−0.18 to 0.76)	0.23
CC	TC or CC	−0.18 (−0.41 to −0.06)	0.036
CG or GG	TC or CC	−0.50 (−0.69 to −0.21)	<0.001

^a^Adjusted for gender, age, smoke and alcohol status, and physical activity.
